# Exogenous PTH 1-34 Attenuates Impaired Fracture Healing in Endogenous PTH Deficiency Mice via Activating Indian Hedgehog Signaling Pathway and Accelerating Endochondral Ossification

**DOI:** 10.3389/fcell.2021.750878

**Published:** 2022-01-05

**Authors:** Cheng Ma, Huan Liu, Yifan Wei, He Li, Dengshun Miao, Yongxin Ren

**Affiliations:** ^1^ Department of Orthopaedics, The First Affiliated Hospital of Nanjing Medical University, Nanjing, China; ^2^ Department of Orthopaedics, The Affiliated Huaian No.1 People’s Hospital of Nanjing Medical University, Huaian, China; ^3^ Nanjing Medical University, Affiliated Friendship Plastic Surgery Hospital, Nanjing, China

**Keywords:** parathyroid hormone, fracture healing, endochondral ossification, chondrocyte, Indian hedgehog signaling pathway

## Abstract

Fracture healing is a complicated, long-term, and multistage repair process. Intermittent administration of parathyroid hormone (PTH) has been proven effective on intramembranous and endochondral bone formation during the fracture healing process, however, the mechanism is unclear. In this study, we investigated the role of exogenous PTH and endogenous PTH deficiency in bone fracture healing and explored the mechanism by using PTH knockout (PTH^-/-^) mice and ATDC5 cells. In a mouse femur fracture model, endogenous PTH deficiency could delay endochondral ossification whereas exogenous PTH promotes accumulation of endochondral bone, accelerates cartilaginous callus conversion to bony callus, enhances maturity of bony callus, and attenuates impaired fracture healing resulting from endogenous PTH deficiency. In fracture callus tissue, endogenous PTH deficiency could inhibit chondrocyte proliferation and differentiation whereas exogenous PTH could activate the IHH signaling pathway to accelerate endochondral ossification and rescue impaired fracture healing resulting from endogenous PTH deficiency. *In vitro,* exogenous PTH promotes cell proliferation by activating IHH signaling pathway on ATDC5 cells. In mechanistic studies, by using ChIP and luciferase reporter assays, we showed that PTH could phosphorylate CREB, and subsequently bind to the promoter of IHH, causing the activation of IHH gene expression. Therefore, results from this study support the concept that exogenous PTH 1-34 attenuates impaired fracture healing in endogenous PTH deficiency mice via activating the IHH pathway and accelerating endochondral ossification. Hence, the investigation of the mechanism underlying the effects of PTH treatment on fracture repair might guide the exploration of effective therapeutic targets for fracture.

## Introduction

Fracture healing is a complicated, long-term, and multistage repair process that is initiated in response to injury, resulting in the generation of new bone and connective tissue with similar anatomy and functionality to the pre-injury site (Meesters et al., 2018). It contains a variety of molecular and cellular events, controlled by numerous, complex cellular signaling pathways, and characterized as partially overlapping sequential phases, which is including inflammation, proliferation, callus formation, and bone remodeling. Firstly, with the periosteum lifted or torn from the bone surface, and the blood vessels broken, a large amount of blood gathers at the fracture ends to form a hematoma, whose coagulation offers a template for callus formation (Einhorn and Gerstenfeld, 2015). As the hematoma develops and nascent blood vessels grow in, mesenchymal stem cells (MSCs) are recruited and differentiated into chondrocytes or osteoblasts simultaneously, initiating intramembranous and endochondral ossification. Osteoblasts are firmly attached to periosteal and endosteal surfaces of fracture ends and ultimately form a “hard callus” covering the entire periphery of the callus. Simultaneously, chondrocytes undergo proliferation and hypertrophy, secrete the cartilage extracellular matrix (ECM), and eventually forms a “soft callus”. Subsequently, the cartilage extracellular matrix undergoes mineralization with chondrocyte apoptosis. The callus is formed by intramembranous and endochondral ossification which is a naive reticulated bone with insufficient hardness and strength gradually cleared by osteoclasts and replaced by lamellar bone structure. Finally, the bone remodeling phase was characterized by the re-established marrow space, the regenerated original marrow structure of hematopoietic tissue, and the original bone structure remodeled as its pre-injury level, which can be considered as a duplicate of embryonic bone development. Most fracture healing processes follow the stages above. According to the characteristics of each stage of fracture healing, various methods and drugs are adopted to accelerate the process of fracture healing (Chen and Luan, 2019; Li et al., 2019; Mu et al., 2019; Nakata et al., 2020).

Parathyroid hormone (PTH), a peptide composed of 84 amino acids, synthesized and secreted by the parathyroid gland plays a critical role in the maintenance of calcium and phosphorus and regulation of bone anabolism and catabolism. Intermittent exposure to PTH stimulates bone formation via promoting proliferation and reducing apoptosis of the osteoblasts whereas continuous exposure to PTH leads to osteoporotic changes via increasing osteoclast activity (Duque et al., 2020). Many studies have found that Intermittent administration of PTH can significantly accelerate the formation of calluses, increase the biomechanical strength of calluses, and promote fracture healing (Nakajima et al., 2002; Alkhiary et al., 2005; Manabe et al., 2007). PTH may have an effect on intramembranous and endochondral bone formation during the fracture healing process (Kakar et al., 2007; Kanezaki et al., 2019; Sun et al., 2020). However, mechanisms of how PTH1–34 affects endochondral ossification during fracture healing remain to be clarified.

Studies have shown that the endochondral bone formation during fracture healing is a recapitulation of endochondral ossification during embryonic and postnatal bone formation in the growth plate (Vortkamp et al., 1998; Ferguson et al., 1999). Hedgehog (HH) signaling pathway is one of the key regulators of embryonic skeletal development, which contains three types of HH proteins in mammals, Sonic HH (SHH), Indian HH (IHH), and Desert HH (DHH). IHH/PTH related protein (PTHrP) feedback loop maintains the number of proliferating chondrocytes, thus maintaining a certain length of the growth plate and maximizing bone growth. IHH^-/-^mice showed a shortened growth plate proliferation zone and accelerated chondrocyte hypertrophy, the same phenotype as PTHrP^-/-^mice (St-Jacques et al., 1999; Karp et al., 2000). Moreover, IHH coordinates chondrocyte proliferation and differentiation independently of PTHrP (Rodda and McMahon, 2006). IHH signaling is also essential in osteoblast differentiation during endochondral bone formation. It has been reported that IHH is required for progenitor cells to differentiate into Runx2-positive osteoblast precursor cells (Rodda and McMahon, 2006). Recent studies have demonstrated that the IHH signaling pathway is involved in fracture healing (Williams et al., 2018; McKenzie et al., 2019).

To investigate whether PTH1-34 enhances fracture healing via activating the Indian hedgehog signaling pathway and how exogenous PTH1-34 impacts the repair processes in the absence of endogenous PTH1-84, we analyzed the callus tissue of 8-week-old wild-type and PTH knockout (PTH^-/-^) mice *in vivo* and explored the mechanism by using an animal model *in vivo* and ATDC5 cells *in vitro* experiments.

## Materials and Methods

### Animals

The PTH knockout (PTH^-/-^) mice (C57BL/6 background) provided in this study had been described previously (Miao et al., 2002). In this study, The PTH^-/-^ mice and their wild-type (WT) littermates were housed in the Experimental Animal Center in Nanjing Medical University under a 12 h/12 h light/dark cycle. All mice were genotyped by using PCR. Food and water were provided *ad libitum* in the cages. Animal use was approved by the Institutional Animal Care and Use Committee of Nanjing Medical University.

### Fracture Model Establishment and Administration of PTH1-34 or Normal Saline

A total of 80 8-week-old mice had standardized mid-diaphyseal femur fracture performed as described previously (Ren et al., 2011). Briefly, by using a mixture of ketamine hydrochloride (100 mg/ml; 80 mg/kg body weight) and xylazine 2% (12 mg/kg body weight) for anesthesia, the left hind leg was shaved and disinfected. A longitudinal incision is made along the long axis of the femur, exposing the distal femur, using a 25G syringe needle to retrogradely insert into the femoral bone marrow cavity until it reaches the proximal end of the femur. After the syringe needle tip is cut and it has been made sure that it is fully seated in the intramedullary space, the incision was sutured with 4-0 nylon sutures. A mid-diaphyseal fracture was created by using three-point bending loading (McBride-Gagyi et al., 2015). Ibuprofen (30 mg/kg/day in their drinking water for 3 days) was used for postoperative analgesia. The mice were divided into four groups (*n* = 20 per group): 1) V-WT, WT mice were administrated with normal saline (NS); 2) V-KO, PTH^-/-^ mice were administrated with NS;3) PTH-WT, WT mice were administrated with PTH 1-34 (3011, R&D, China) (50 μg/kg/d, mouse body weight) subcutaneously; 4) PTH-KO, PTH^-/-^ mice were administrated with PTH1-34 (50 μg/kg/d) subcutaneously. Daily subcutaneous injections of NS or hPTH1-34 (50 μg/kg/d) were administered subcutaneously from the postoperative day 1 and continued for 28 days post-fracture. The mice were sacrificed and the fractured femurs were harvested at postoperative 7, 14, 21, and 28 days for further examinations (For each group at one timepoint, *n* = 5).

### Radiological Examination

After the mice were sacrificed, the fractured femurs whose soft tissue was removed received X-ray and micro-CT examination. Contact radiographs were taken using a Faxitron model 805 radiographic inspection system (Faxitron Contact, Faxitron, Germany) (22 kV voltage and 4 min exposure time). X-Omat TL film (Eastman Kodak Co., Rochester, NY, United States) was used and processed routinely. Femurs were analyzed by micro-CT with a SkyScan 1072 scanner and associated analysis software (SkyScan, Antwerp, Belgium) as described below. Briefly, image acquisition was performed at 100 kV and 98 mA with a 0.98 rotation between frames. During scanning, the samples were enclosed in tightly fitting plastic wrap to prevent movement and dehydration. Thresholding was applied to the images to segment the bone from the background. Two-dimensional images were used to generate three-dimensional renderings using the 3D Creator software supplied with the instrument. The resolution of the micro-CT images is 18.2 mm. The region of interest (ROI) was defined at the center of fracture, from the proximal to the distal end of the callus region. Total tissue volume (TV, mm^3^), callus volume (CV, mm^3^), bone volume (BV, mm^3^), bone volume fraction (BV/TV), bone mineral density of callus (BMD, mg HA/cm^3^), Trabecular number (Tb.N, mm), Trabecular thickness (Tb.Th, mm), and Trabecular separation (Tb.Sp, mm) were calculated.

### Histology and Immunohistochemistry

The left femurs were fixed in 4% paraformaldehyde (PFA) solution overnight at 4°C, decalcified in EDTA-glycerol solution for 14–21 days at 4°C, dehydrated, hyalinized, immersed in wax, embedded in paraffin and sectioned into 5-μm-thick slices for histological staining or immunohistochemistry (IHC). The dewaxed and rehydrated sections were stained with hematoxylin and eosin (H&E, Jiancheng, Nanjing, China) and safranin-O/fast green (G1371, Solarbio, Beijing, China) according to the instructions of the manufacturer.

Briefly, after deparaffinization and hydration, sections were immersed in sodium citrate (10 mM, >92°C) for 8 min (antigen retrieval) and incubated with 3% hydrogen peroxide in the dark for 30 min (endogenous peroxidase inactivation). Then, 10% goat serum was used for blocking the non-specific binding of antibodies at room temperature for 30 min. The primary antibody dilution solution was applied for incubation overnight at 4°C. Following by rinsing with phosphate-buffered saline (PBS) for 3 times, sections were chronologically incubated: in biotin-conjugated secondary antibodies for 1 h at room temperature, Elite ABC (PK6100, Vector, United States) for 1 h at room temperature, and diaminobenzidine (DAB, SK4100, Vector, United States) until the expected stain intensity was reached. After washing with PBS, the sections were counterstained with hematoxylin and observed by using a microscope. All images were photographed using Leica LAS-X software under Leica DMi8 microscope. The images of IHC staining were analyzed with Image-Pro Plus. The images of safranin-O/fast green staining were analyzed using ImageJ software (NIH). The list of primary and second antibodies is shown in Supplementary Table S1.

### Western Blot

Total proteins were extracted from fracture callus tissue or ATDC5 cells by using a Protein Extraction Kit (P0013, Beyotime, Shanghai, China) and the concentration was measured by a BCA Protein Assay Kit (P0012S, Beyotime, Shanghai, China). Western blotting was performed according to a previous study (Wang et al., 2019). Equal amounts of total protein samples were separated by sodium dodecyl sulfate-polyacrylamide gel electrophoresis gels and transferred to polyvinylidene difluoride (PVDF) membranes. After being blocked with 5% skim milk solution and then incubated with diverse primary antibodies at 4 °C overnight, the membranes were incubated with horseradish peroxidase (HRP)-conjugated goat anti-mouse or goat-anti-rabbit antibodies (KPL, United States). Bands were visualized using ECL Western blotting Detection kit (P0018S, Beyotime, Shanghai, China) and analyzed using ImageJ software (NIH, United States). The list of primary and secondary antibodies is shown in Supplementary Table S2.

### Quantitative Real-Time Reverse-Transcription-PCR

Total RNA was collected from fracture callus tissue or ATDC5 cells by using TRIzol reagent (15596026, Invitrogen, Shanghai, China) and subsequently was applied to reverse transcription to cDNA by using HiScript III Reverse Transcriptase (R302, Vazyme, Nanjing, China). The qRT-PCR was performed by using the ABI 7500 Fast Real-Time PCR System (Applied Biosystems). The expression level of target genes was normalized to GAPDH by using the comparative 2^-ΔΔ^ Ct method. The qRT-PCR primer sequences are shown in Supplementary Table S3.

### Cell Culture

ATDC5 cells were cultured as monolayer adherent cells in a complete medium containing Dulbecco’s modified Eagle’s medium and Ham’s F-12 medium (DMEM/F12, 11320033, Gibco, Shanghai, China), 10% fetal bovine serum (FBS, GE Healthcare), and 100 U/mL penicillin-streptomycin (15070063, Gibco, Shanghai, China). At 80%–90% confluency, cells were passaged using trypsin, seeded in various culture plates, and maintained in an incubator with 5% CO_2_ at 37°C. PTH1-34 was dissolved in NS and cyclopamine in Dimethyl sulfoxide (DMSO); the same volume of NS or DMSO was used as the control. Different concentrations of PTH1-34 (0, 10^−11^, 10^−10^, 10^−9^, 10^−8^, and 10^−7^ mol/L), NS, DMSO, or cyclopamine (hedgehog signaling pathway antagonist, S1146, Selleck Chemicals, Shanghai, China) were added according to the group. Cell cultures were divided into four groups; 1) CON, the cells were treated with Dimethyl sulfoxide (DMSO); 2) PTH, the cells were only treated with PTH1-34;3) CYC, the cells were only treated with cyclopamine; 4) PTH+CYC, the cells were subjected to PTH 1-34 and simultaneously treated with cyclopamine. All types of culture medium were maintained for 6 h per 24 h, then changed to complete medium.

### Cell Viability Assay

To assess the effect of PTH1-34, ATDC5 cells were cultured in 96-well multiplates at 5 × 10^3^cells per well, followed by incubation with different concentrations of PTH1-34 or the same volume of NS for 6 h per 24 h after cell attachment. Cell Counting Kit-8 (Dojindo, Kumamoto, Japan) was applied to measure the absorbance at day 1–6 timepoints using SpectraMaxM (Molecular Devices, San Jose, CA, United States), according to the manufacturer’s instructions.

### Toluidine Blue Staining and Alcian Blue Staining

The ATDC5 cells were fixed by 4% formaldehyde in the 24-well plates for 15 min. After rinsing with PBS 3 times, the cells were permeabilized with toluidine blue working solution (Toluidine blue O 0.01 g, 70% alcohol 1 ml, 1% Sodium chloride solution 9 ml) and Alcian Blue Cartilage Stain solution (G2541, Solarbio, Beijing, China). The stained cells were photographed using Leica LAS-X software under Leica DMi8 fluorescent microscope.

### Immunofluorescence

After PBS washing 3 times, 4% formaldehyde was used to fix the ATDC5 cells cultured in the 24-well plates for 15 min. The cells were permeabilized with 0.5% Triton X-100 for 30 min, then blocked with 5% bovine serum albumin (BSA, Sigma, Ohio, United States) -PBS for 1 h at room temperature. The primary antibody dilution solution was applied for incubation overnight at 4°C. Following by rinsing with PBS 3 times, the cells were chronologically incubated with the appropriate secondary antibody for 2 h at room temperature in the dark and counterstained with DAPI (SouthernBiotech, Alabama, United States) for 5 min at room temperature in the dark. All images were photographed using Leica LAS-X software under Leica DMi8 fluorescent microscope and analyzed using ImageJ software (NIH). The list of primary and second antibodies is shown in Supplementary Table S4.

### Luciferase Reporter Assay

The promoter region of the hedgehog gene was defined as within the 2000-bp region upstream according to the National Center for Biotechnology Information database (http://www.ncbi.nlm.nih.gov/). JASPAR core database (Fornes et al., 2020) was used to predict DNA-binding sites for cAMP-response element-binding protein (CREB) transcription factor in the IHH promoter. Then, the cells were transfected with the two types of vectors combined with Renilla vector (Promega Corp., Madison, Wisconsin, United States) and mutate or negative controls, respectively. The luciferase was detected by use of luciferase reporter assay reagents (Promega Corp., Madison, Wisconsin, United States).

### Chromatin Immunoprecipitation

ChIP assay was performed using ATDC5 cells, and the specific steps were followed according to the manufacturer’s instructions (17-610, Millipore, United States). Briefly, cells were consecutively crosslinked by incubation in 1% formaldehyde-containing medium for 10 min at room temperature, sonicated to an average fragment size of 500 bp, and centrifuged to remove cell debris. A small portion (10%) of each sample was removed as an input control, and anti-p-CREB (1:50, 9198, Cell Signaling Technology, United States) and the rabbit normal IgG antibody (5 µl, 2729, Cell Signaling Technology, United States) was added to the remaining samples. The protein–DNA complex was collected with protein A-Sepharose beads, and then eluted and reverse cross-linked. DNA was recovered using a PCR purification kit (Qiagen, Hilden, Germany) and assessed by real-time PCR.

### Statistical Analysis

All data were expressed as the mean ± standard deviation (SD) and analyzed by SPSS software (version 20.0, United States). One-way analysis of variance (ANOVA) followed by Student–Newman–Keuls (SNK) post hoc test was adopted for multiple groups comparison. An independent *t*-test was adopted for a two-group comparison. All graphs were presented using GraphPad Prism Software (version 8.0.0, United States). *p* < 0.05 was considered to indicate a statistically significant difference.

## Results

### The Role of Endogenous PTH Deficiency and Exogenous PTH on Cartilaginous and Bony Callus Formation

**FIGURE 1:**
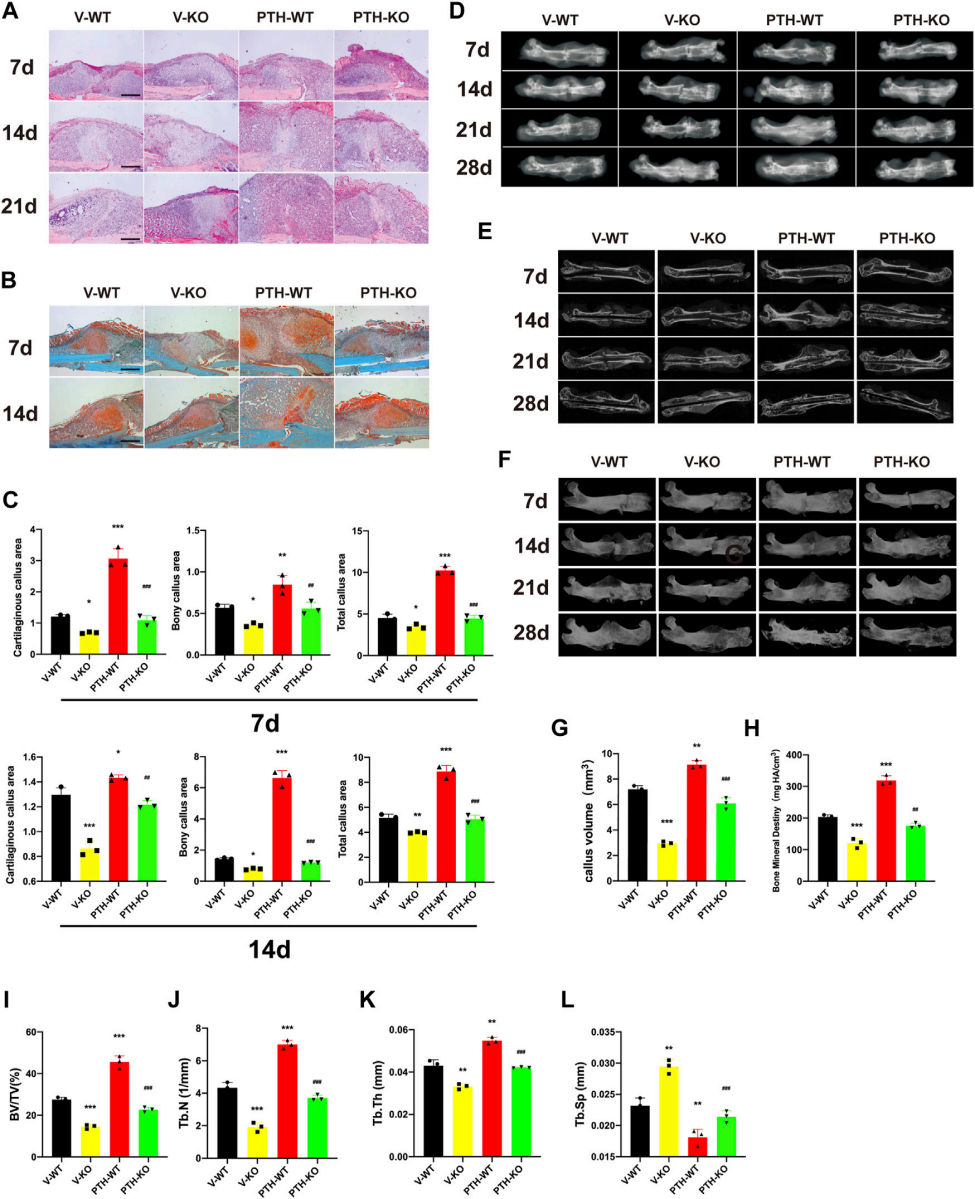
The role of endogenous PTH deficiency and exogenous PTH on cartilaginous and bony callus formation. **(A)** Representative images of H&E staining of paraffin callus sections from V-WT, V-KO, PTH-WT, and PTH-KO groups at 7, 14, 21 days post-fracture, Scale bar: 50 μm. **(B)** Representative images of Safranin-O/fast green staining of paraffin callus sections from V-WT, V-KO, PTH-WT, and PTH-KO groups at 7, 14, 21 days post-fracture, Scale bar: 50 μm. **(C)** Representative radiographs of fractured femurs from V-WT, V-KO, PTH-WT, and PTH-KO groups at 7, 14, 21, and 28 days post-fracture. **(D)** Quantitative assessment of cartilaginous callus area, bony callus, and total callus area of V-WT, V-KO, PTH-WT, and PTH-KO groups at 7 and 14 days post-fracture. **(E,F)** Representative longitudinal micro-CT and 3D reconstructions images of the fractured femurs from V-WT, V-KO, PTH-WT, and PTH-KO groups at 7, 14, 21, and 28 days post-fracture. Quantitative assessment of fracture callus of V-WT, V-KO, PTH-WT, and PTH-KO groups at 14 days post-fracture. **(G)** callus volume **(H)** bone mineral density **(I)** bone volume/total volume **(J)** trabecular number **(K)** trabecular thickness **(L)** trabecular separation. All data are expressed as mean ± SD (*n* = 3). (*p < 0.05; **p < 0.01; ***p < 0.001; compared with control group. ^#^ *p* < 0.05; ^##^ *p* < 0.01; ^###^ *p* < 0.001; compared with V-KO group).

To explore the effects of endogenous PTH deficiency and exogenous PTH on callus formation, fractured femurs harvested at days 7, 14, 21, and 28 were examined by radiography. H&E staining and radiographs revealed a visible increase in callus size and apparent mineralized callus visible by day 14 in the PTH-WT group, whereas there was less apparent mineralized callus in the V-KO group compared to the V-WT group. In the PTH-KO group, the visible mineralized callus was larger than that in the V-KO group, with no visible differences compared to V-WT groups. At days 28 post-fracture, although the external calluses in both groups were fully bridged, the interior callus appeared denser in the V-KO group, while this region was more radiolucent in the PTH-WT group. There were no visible differences between the V-WT and PTH-KO groups, but calluses in both were more radiolucent than those in V-KO group (Figures 1A,D).

Safranin-O/fast green staining indicated that total callus areas, cartilaginous callus areas, and bony callus areas in PTH-WT were increased significantly whereas those in the V-KO group were decreased significantly at days 7 and 14 post-fracture, compared to the V-WT group. In addition, those measurements in the PTH-KO group were obviously higher than those in the V-KO group at days 7 and 14 post-fracture (Figures 1B,C).

To further evaluate callus formation, fractured femurs harvested at days 7, 14, 21, and 28 were examined by micro-CT. As shown in Figures 1E,F, representative 3D reconstructions and micro-CT images indicated that the PTH-WT group appears a larger callus size whereas the V-KO group appears a smaller size, while external callus size in the V-WT group and PTH-KO groups was similar between the size in the PTH-WT group and the V-KO group, which is consistent with radiographic observations. The quantitative assessment showed that the callus volume, BMD, BV/TV, Tb.N, Tb.Th in PTH-WT was increased significantly whereas those in PTH-KO group were decreased significantly at days 14 post-fracture, compared to the V-WT group. In addition, those measurements in the PTH-KO group were obviously higher than those in the V-KO group at days 14 post-fracture. The Tb.Sp in the PTH-WT group was decreased significantly whereas those in the V-KO group were increased significantly at days 14 post-fracture, compared to the V-WT group. Additionally, those measurements in PTH-KO group were obviously lower than those in the V-KO group at days 14 post-fracture, suggesting that intermittent PTH 1–34 treatment was able to rescue callus formation in PTH^-/-^mice and significantly enhance callus formation in all genotyped mice (Figures 1G–L).

Taken together, these data indicated that endogenous PTH deficiency could delay endochondral ossification whereas exogenous PTH could promote the accumulation of endochondral bone, accelerate cartilaginous callus conversion to bony callus, enhance the maturity of bony callus and attenuate impaired fracture healing in endogenous PTH deficiency mice.

### The Role of Endogenous PTH Deficiency and Exogenous PTH on Chondrocyte Proliferation and Differentiation at the Callus

To explore the effects of endogenous PTH deficiency and exogenous PTH on chondrocyte proliferation and differentiation at the callus, the messenger RNA (mRNA) and proteins expression of Col Ⅱ, Col Ⅹ, PTHR1, PCNA, and p-CREB were examined by qRT-PCR, Western Blot, and IHC staining.

**FIGURE 2:**
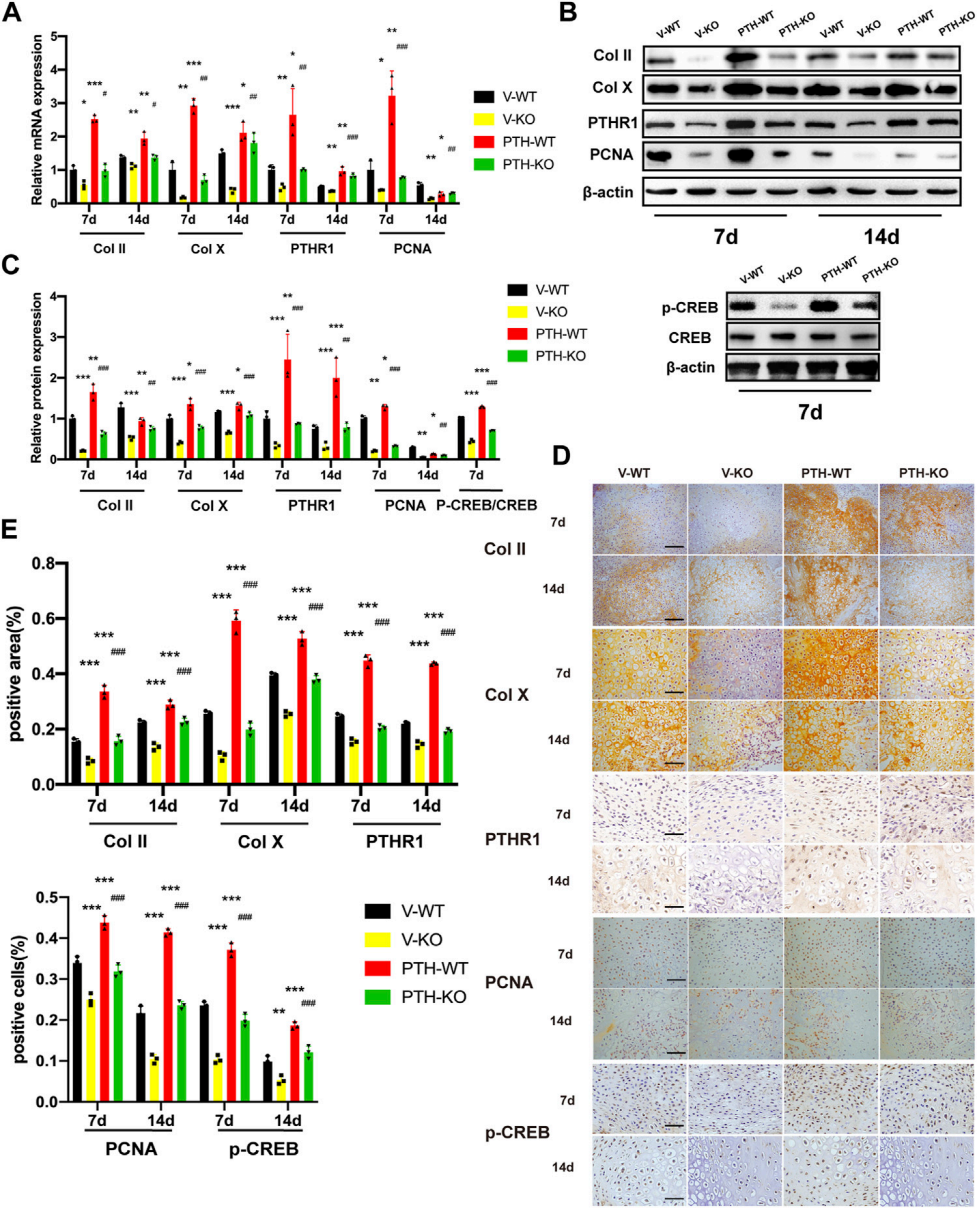
The role of endogenous PTH deficiency and exogenous PTH on chondrocyte proliferation and differentiation at the callus. (**A)** The qRT-PCR analysis of Col Ⅱ, Col X, PTHR1 and PCNA mRNA expression in V-WT, V-KO, PTH-WT, and PTH-KO group at 7 and 14 days post-fracture. GAPDH was used for normalization (*n* = 3/group). **(B,C)** Western blot analysis of the IHH, SMO, Gli1, and Gli3 protein expression in V-WT, V-KO, PTH-WT, and PTH-KO group at 7 and 14 days post-fracture and the p-CREB and CREB protein expression at 7 days post-fracture. *β*-Actin was used for normalization (*n* = 3/group). **(D)** Representative images of IHC staining of paraffin callus sections for Col Ⅱ, Col X, PTHR1, p-CREB, and PCNA from V-WT, V-KO, PTH-WT, and PTH-KO mice at 7 and 14 days post-fracture, Scale bar: 25 μm. **(E)** Quantitative analysis of IHC staining. All data are expressed as mean ± SD (*n* = 3). (*p < 0.05; **p < 0.01; ***p < 0.001; compared with control group. ^#^ *p* < 0.05; ^##^ *p* < 0.01; ^###^ *p* < 0.001; compared with V-KO group).

At day 7 post-fracture, the mRNA expression of Col Ⅱ, Col Ⅹ, PTHR1, and PCNA in the PTH-WT group was increased significantly whereas those in the V-KO group were decreased significantly, compared to the V-WT group. In addition, those indicators in the PTH-KO group were obviously higher than those in the V-KO group. At 14 days post-fracture, the mRNA expression of Col Ⅱ and Col Ⅹ, and PTHR1 in the PTH-WT group were increased significantly whereas those in the V-KO group were decreased significantly, compared to the V-WT group. In addition, those indicators in the PTH-KO group were obviously higher than those in the V-KO group. Interestingly, the mRNA expression of PCNA was decreased dramatically at 14 days post-fracture, compared with the V-WT group (Figure 2A).

Western blot and IHC analysis showed that the protein expression of Col Ⅱ, Col Ⅹ, PTHR1, and PCNA exhibited the same tendency consistent with results in qRT-PCR analysis. At 7 days post-fracture, the expression of p-CREB proteins was remarkably increased in the PTH-WT group and decreased in the V-KO group compared with the V-WT group while the expression in the PTH-KO group was obviously higher than those in the V-KO group (Figures 2B–E).

Taken together, these data indicated that endogenous PTH deficiency could inhibit chondrocyte proliferation and differentiation at the callus whereas exogenous PTH could phosphorylate CREB, promote chondrocyte proliferation and attenuate impaired fracture healing resulting from endogenous PTH deficiency.

### The Role of Endogenous PTH Deficiency and Exogenous PTH on the Regulation of Indian Hedgehog Signaling Pathway at the Callus

To explore the effects of endogenous PTH deficiency and exogenous PTH regulation of Indian hedgehog signaling pathway at the callus chondrocyte proliferation and differentiation at the callus, the expression of IHH, Smo, Gli1, and Gli3 were examined by qRT-PCR, Western Blot, and IHC staining.

At day 7 post-fracture, the mRNA expression of IHH, Smo, and Gli1 in the PTH-WT group were increased significantly whereas the mRNA expression in the V-KO group were decreased significantly, compared to the V-WT group. In addition, those indicators in the PTH-KO group were obviously higher than those in the V-KO group. Conversely, the mRNA expression of Gli3 in the PTH-WT group was decreased significantly whereas the mRNA expression in the V-KO group was increased significantly, compared to the V-WT group. Moreover, the mRNA expression in the PTH-KO group was obviously lower than those in the V-KO group.

**FIGURE 3:**
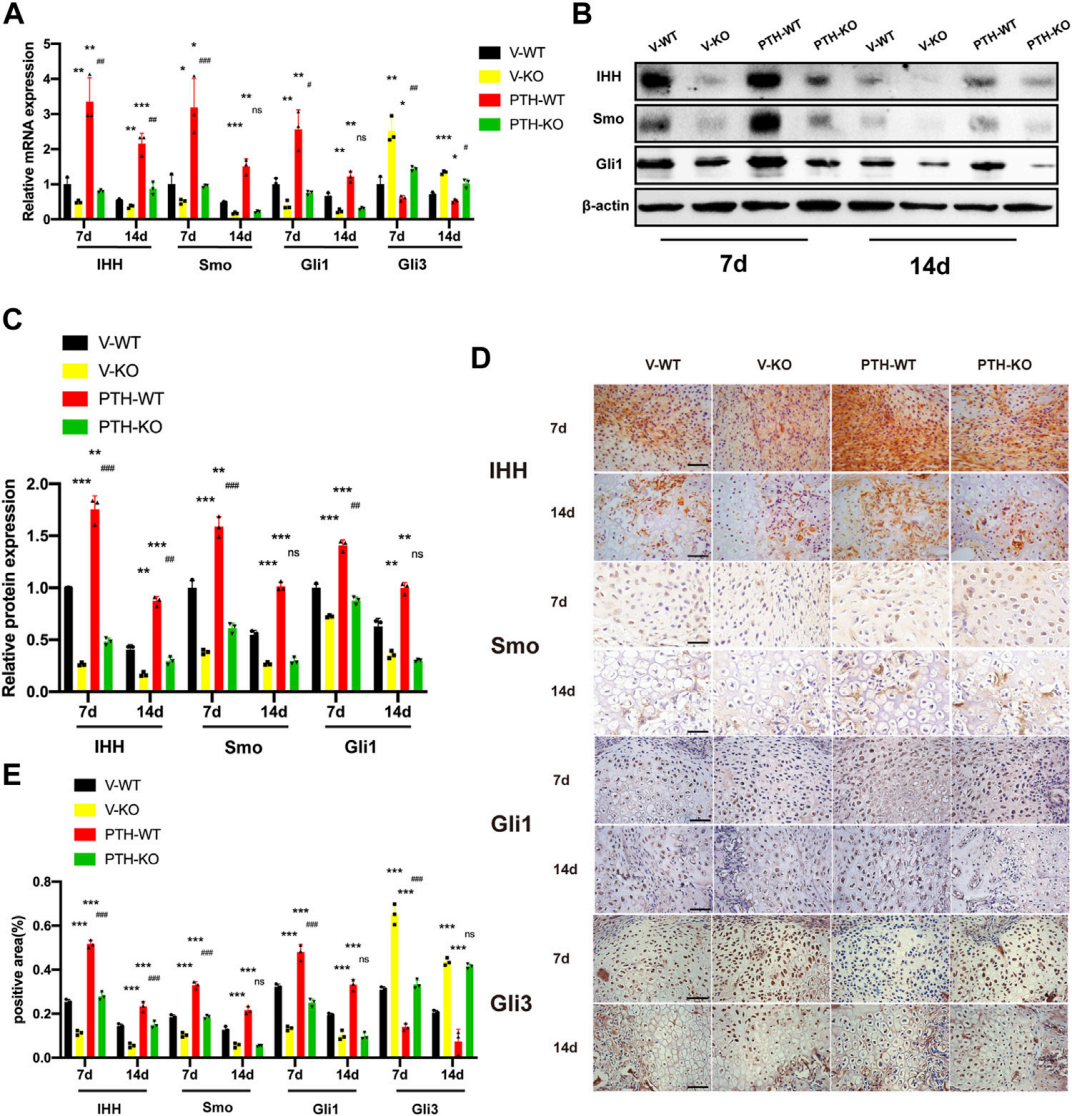
The role of endogenous PTH deficiency and exogenous PTH on the regulation of Indian hedgehog signaling pathway at the callus. **(A)** The qRT-PCR analysis of IHH, SMO, Gli1, and Gli3 mRNA expression in V-WT, V-KO, PTH-WT, and PTH-KO groups at 7 and 14 days post-fracture. GAPDH was used for normalization (*n* = 3/group). **(B,C)** Western blot analysis of the IHH, SMO, and Gli1 protein expression in V-WT, V-KO, PTH-WT, and PTH-KO groups at 7 and 14 days post-fracture. *β*-Actin was used for normalization (*n* = 3/group). **(D)** Representative images of IHC staining of paraffin callus sections for IHH, SMO, Gli1, and Gli3 from V-WT, V-KO, PTH-WT, and PTH-KO groups at 7 and 14 days post-fracture, Scale bar: 25 μm. **(E)** Quantitative analysis of IHC staining. All data are expressed as mean ± SD (*n* = 3). (*p < 0.05; **p < 0.01; ***p < 0.001; compared with control group. ^#^ *p* < 0.05; ^##^ *p* < 0.01; ^###^ *p* < 0.001; compared with V-KO group).

At day 14 post-fracture, the mRNA expression of IHH, Smo, and Gli1 in the PTH-WT group were increased significantly whereas the mRNA expression in the V-KO group was decreased significantly, compared to the V-WT group. In addition, the mRNA expression of IHH in the PTH-KO group was obviously higher than that in the V-KO group. However, the mRNA expression of Smo and Gli1 were not significantly different between the V-KO and PTH-KO groups. Conversely, the mRNA expression of Gli3 in the PTH-WT group was decreased significantly whereas the mRNA expression in the V-KO group was increased significantly, compared to the V-WT group. Moreover, the mRNA expression in the PTH-KO group was lower than that in the V-KO group (Figure 3A). Western blot and IHC analysis showed that the protein expression of IHH, Smo, and Gli1 exhibited the same tendency consistent with results in qRT-PCR analysis (Figures 3B–E).

Collectively, these data indicated that endogenous PTH deficiency could suppress IHH signaling pathway whereas exogenous PTH could activate IHH signaling pathway to accelerate endochondral ossification and almost completely rescue the inhibition of the IHH signaling pathway resulting from endogenous PTH deficiency.

### The Exogenous PTH Promotes Proliferation by Activating IHH Signaling Pathway on ATDC5 Cells

**FIGURE 4:**
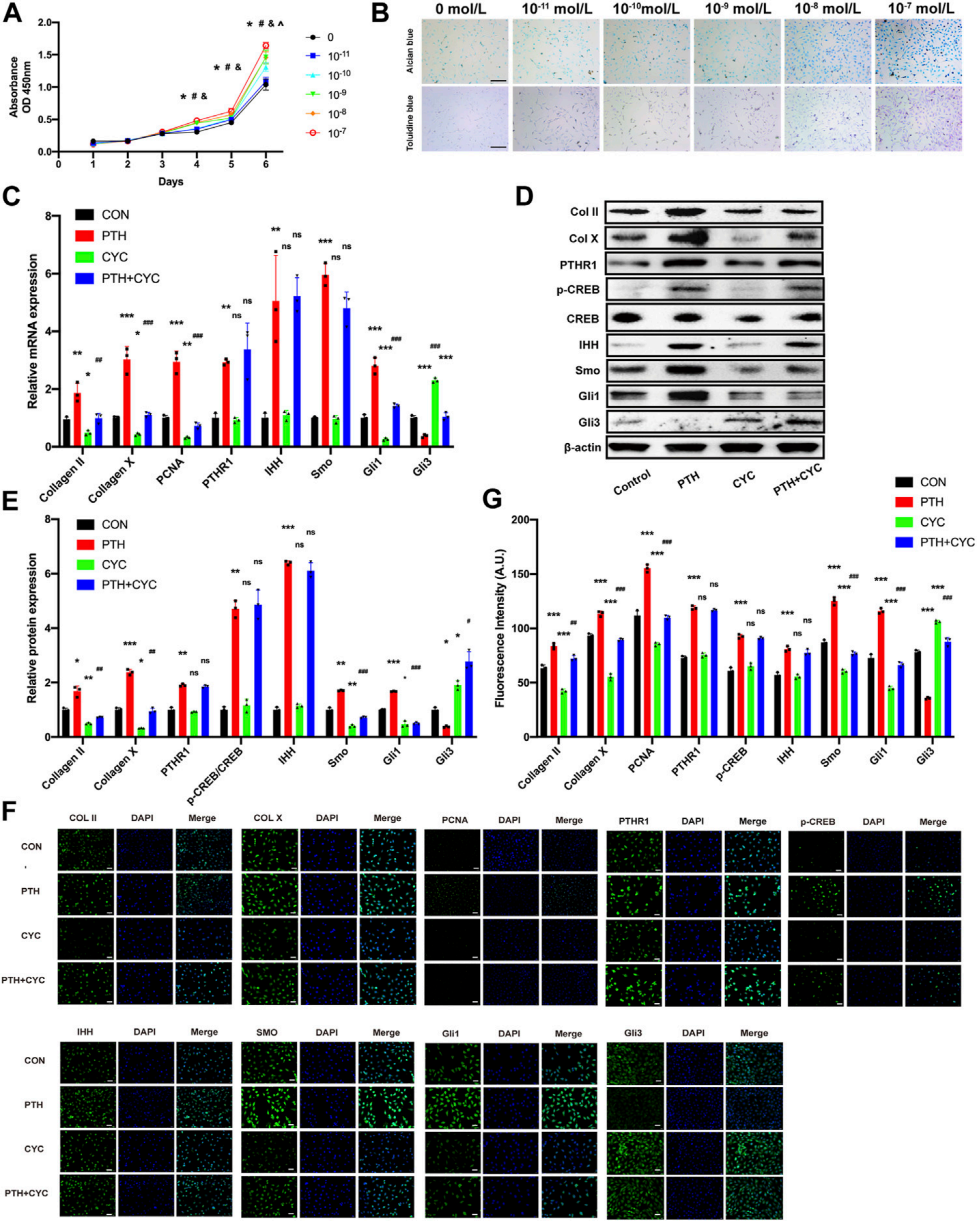
The exogenous PTH promotes proliferation by activating IHH signaling pathway on ATDC5 cells. **(A)** Growth curves of ATDC5 cells at different concentrations of PTH1-34 measured by CCK8-assay (n = 6/group) (*p < 0.05, 0 mol/L vs. 10^−7^ mol/L, ^#^ *p* < 0.05, 0 mol/L vs. 10^−8^ mol/L, ^&^ *p* < 0.05, 0 mol/L vs. 10^−9^ mol/L, ^^^ *p* < 0.05, 0 mol/L vs. 10^−10^ mol/L). **(B)** Toluidine blue staining and Alcian blue staining in ATDC5 cells at days 7 after incubation with different concentrations of PTH1-34, Scale bar: 50 μm. **(C)** The qRT-PCR analysis of Col Ⅱ, Col X, PCNA, PTHR1, IHH, SMO, Gli1, and Gli3 mRNA expression in the groups at day 4 in the presence or absence of PTH (10^−7^ mol/L) and cyclopamine (10^−6^ mol/L). GAPDH was used for normalization (*n* = 3/group). **(D,E)** Western blot analysis of the Col Ⅱ, Col X, PTHR1, p-CREB, CREB, IHH, SMO, Gli1, and Gli3 protein expression in the groups at day 4 in the presence or absence of PTH (10^−7^ mol/L) and cyclopamine (10^−6^ mol/L). *β*-Actin was used for normalization (*n* = 3/group). **(F)** Representative IF staining images of Col Ⅱ, Col X, PCNA, PTHR1, p-CREB, IHH, Smo, Gli1, and Gli3 expression levels in ATDC5 cells in the groups at days 4 in the presence or absence of PTH (10^−7^ mol/L) and cyclopamine (10^−6^ mol/L), Scale bar: 20 μm. **(G)** Quantitative analysis of IF staining. All data are expressed as mean ± SD (*n* = 3). (*p < 0.05; **p < 0.01; ***p < 0.001; compared with control group. ^#^ *p* < 0.05; ^##^ *p* < 0.01; ^###^ *p* < 0.001; compared with PTH group).

To investigate the potential effect of PTH on proliferation, we cultured ATDC5 cells with different concentrations of PTH1-34 (0, 10^−11^, 10^−10^, 10^−9^, 10^−8^, and 10^−7^ mol/L) for 6 days. The CCK-8 assays demonstrated that all treatment groups (10^−11^, 10^−10^, 10^−9^, 10^−8^, 10^−7^, and 0 mol/L) showed no significant difference for the first 3 days. At days 4 and 5, three treatment groups (10^−9^, 10^−8^, and 10^−7^ mol/L) exhibited the enhanced cell viability in a concentration-dependent manner, compared to the Control group. Moreover, by day 6, four treatment groups (10^−10^,10^−9^, 10^−8^, and 10^−7^ mol/L) exhibited enhanced cell viability in a concentration-dependent manner, compared to the control group (Figure 4A). Further, the intensities of toluidine blue and Alcian blue staining were higher in the treatment groups at day 4, especially at a concentration of 10^−8^ or 10^−7^ mol/L, suggesting that exogenous PTH could increase the formation of cartilage-specific proteoglycan (Figure 4B). Therefore, these data indicated that exogenous PTH could promote ATDC5 cells proliferation.

To investigate whether exogenous PTH could activate IHH signaling pathway to promote cell proliferation, we stimulated ATDC5 cells in the presence or absence of PTH (10^−7^ mol/L) and cyclopamine (10^−6^ mol/L) for 4 days, followed by qRT-PCR analysis, Western blot, and IF staining.

The qRT-PCR analysis indicated that the mRNA expression of Smo, Gli1, PCNA, Col Ⅱ, and Col Ⅹ in the PTH group was increased obviously whereas the expression in the CYC group was decreased significantly, compared to the Control group. Additionally, the expression above-mentioned in the PTH&CYC group was lower than that in the PTH group. On the contrary, the mRNA expression of Gli3 in the PTH group was decreased obviously whereas the expression in the CYC group was increased significantly, compared to the Control group. Moreover, the expression in the PTH&CYC group was higher than that in the PTH group. Interestingly, the mRNA expression of PTHR1 and IHH in the PTH group was increased obviously, however, there were no significant differences between the Control group and CYC group as well as between the PTH group and PTH&CYC group (Figure 4C). Western blot and IHC analysis showed that the protein expression of PTHR1, p-CREB, IHH, Smo and Gli1, Gli3, PCNA, Col Ⅱ, and Col Ⅹ exhibited the same tendency consistent with results in qRT-PCR analysis (Figures 4D–G).

Taken together, these data demonstrated that exogenous PTH could activate the IHH signaling pathway to promote cell proliferation on ATDC5 cells while cyclopamine could almost completely impair the effect of PTH treatment in cell proliferation promotion and in IHH signaling pathway activation.

### The Transcription Factor CREB Facilitates IHH Expression Through Promoter Activation

**FIGURE 5:**
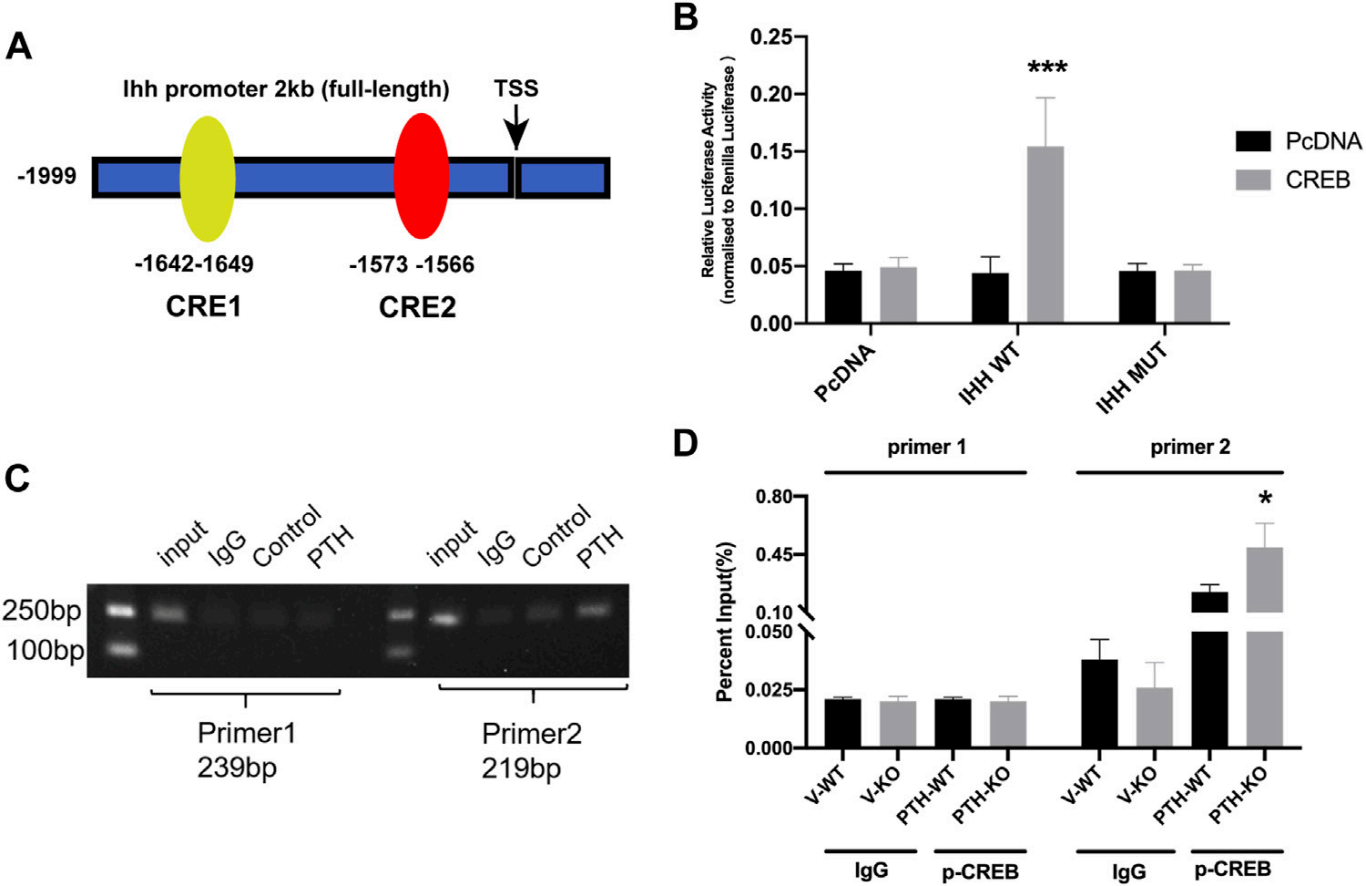
The transcription factor CREB facilitates IHH expression through promoter activation. **(A)** A diagram shows the relative positions of full-length (FL) and fragments of IHH promoter reporters. **(B)** The dual-luciferase assays show that overexpression of CREB significantly increased the luciferase activity of WT-IHH but not MUT-IHH. **(C,D)** ChIP-qPCR shows that PTH could phosphorylate CREB and directly bind to −1642 to −1649 bp of IHH promoter. All data are expressed as mean ± SD (*n* = 3). (*p < 0.05; **p < 0.01; ***p < 0.001, compared with control group).

To elucidate the mechanism of the effect of PTH on IHH expression, we utilized JASPAR and TFSEARCH online software to predetermine the transcription factor of IHH. CREB obtained the highest predicting score, and both prediction software programs pointed it, as one of the potential transcription factors of the IHH promoter region (Figure 5A). ChIP and luciferase reporter assays were performed. The results not only indicated that CREB could actually bind to the promoter of IHH on -1642∼-1649 bp, but also showed that PTH phosphorylates CREB, which directly resulted in an increase of IHH promoter activity (Figures 5B–D). Taken together, these data strongly testified that PTH could phosphorylate CREB, and subsequently bind to the promoter of IHH, causing the activation of IHH gene expression.

## Discussion

In this study, we demonstrated that both endogenous PTH deficiency and exogenous PTH cause a dramatic change of molecular and cellular events in the endochondral ossification process associated with bone fracture healing. Endogenous PTH deficiency results in smaller total callus volume and lowers BV/TV at 14 days post-fracture. Downregulation of the cartilaginous callus formation gene and protein expression was observed, of which Col II, PCNA, and Col X expression significantly decreased at days 7 and 14 post-fracture. These results indicate that endogenous PTH deficiency delays endochondral ossification during fracture healing via affecting chondrocyte proliferation and hypertrophy at the callus, corresponding with our previous study (Ren et al., 2011). We previously reported that PTH^-/-^ mice present poor capillary invasion, mineralized cartilage matrix reduction, and osteoblast diminution that led to a poor primary spongiosa development and ultimately to trabecular bone volume reduction (Miao et al., 2002). Moreover, endogenous PTH deficiency affected osteoclast activity during fracture healing and resulted in delayed endochondral ossification (Sun et al., 2020). Angiogenic capacity was also decreased in the PTH^-/-^ femoral fracture mice, which affects endochondral bone formation, thereby resulting in delayed fracture healing. Therefore, regarding the fracture repair process as a duplicate of embryonic bone development, endogenous PTH plays a critical role in endochondral bone formation during the fracture healing process. In several studies, it has been shown that exogenous administration of PTH1-34 or PTH1-84 PTH improves the fracture healing process. Exogenous PTH1-34 resulted in increased new bone formation, improvements in bone mechanical strength, bone mineral content (BMC), external callus volume, and callus strength as well as accelerated remodeling and earlier replacement of woven bone by lamellar bone in animal models (Andreassen et al., 1999; Alkhiary et al., 2005; Mognetti et al., 2011).In addition, similar biological effects were observed in complicated fracture animal models, including osteoporotic fractures, diabetes, stress fractures, open fractures, and nonunion (Jahng and Kim, 2000; Tagil et al., 2010; Lin et al., 2012; Hamann et al., 2014). In clinic, there are several case reports to support the use of PTH1-34 or PTH 1-84 for fracture healing in humans (Zhang et al., 2014). Our results from this study also showed that Col II, Col X, and PCNA expression by using qRT-PCR, Western blot, and IF staining were significantly upregulated at days 7 and 14 post-fracture, suggesting that exogenous PTH1-34 promotes chondrocyte differentiation and hypertrophy at callus. Moreover, using radiographic imaging analysis and histology, increased total callus volume, higher BV/TV and enhanced BMD, larger bony callus area in total callus are observed in the PTH-WT group, suggesting that not only enhance the formation of cartilage and bony callus, but also accelerate the transformation of cartilage callus into bony callus as well as endochondral ossification. We also found that upregulation gene and protein expression associated with chondrocyte proliferation, hypertrophy and differentiation and accelerated radiographic alterations related to callus morphologic transformation were obtained in PTH-KO group, compared with V-KO group. These findings indicated that exogenous PTH1-34 partially ameliorated delayed endochondral ossification in PTH^-/-^ mice models, which was consistent with previous studies (Kakar et al., 2007; Ren et al., 2011). The ATDC5 cell line used in this study derived from mouse embryonal calcinoma (EC) reflect the complete process of chondrocyte proliferation, differentiation and mineralization in endochondral ossification, which has been widely accepted. Intermittent PTH leads to positive effects on cellular proliferation, proteoglycan distribution, and mineralization of mandibular condylar cartilage in cell culture ^(^ MH et al., 2017 ^)^. The results presented here show that PTH1-34 especially at concentrations of 10^−7^ mol/L significantly improved the cell viability by using CCK-8 assays, elevated Col II, Col X and PCNA expression by using qRT-PCR, Western blot and IF staining, indicating that PTH1-34 might be able to promote cells proliferation.

Although these findings may prove that exogenous PTH1-34 accelerates endochondral ossification and partially ameliorates delayed endochondral ossification in endogenous PTH deficiency mice models via promoting chondrocyte cell proliferation and differentiation, the mechanism needs to be further elucidated. Hedgehog signaling pathway play an important role in endochondral bone formation. The IHH/PTHrP feedback loop regulated growth plate chondrocytes proliferation and differentiation for normal skeletal development has been demonstrated by a variety of experimental strategies. Meanwhile, IHH stimulates chondrocytes proliferation and differentiation in a manner independent of PTHrP. During fracture healing, local administration of a hedgehog agonist resulted in an increase in chondrocyte proliferation in cartilaginous callus and the promotion of bone formation in bony callus in a mouse model (Kashiwagi et al., 2016). It has been reported that the administration of PTH promoted proliferation by regulating the expression of PTHrP and IHH at days 2, 3 post-fracture, and chondrocyte maturation was rapidly enhanced at days 5 post-fracture (Kakar et al., 2007). PTH may increase the aggregation of chondrocytes in the callus and accelerate its maturation and mineralization by affecting the PTHrP-IHH feedback loop and canonical Wnt signaling pathway (Kakar et al., 2007). In this study, we observed that IHH mRNA and its protein expression dramatically increased in the PTH-WT group and decreased in the V-KO group at 7 and 14 days post-fracture compared to the V-WT group, and simultaneously increased in PTH-KO compared to the V-KO group, indicating that endogenous PTH deficiency downregulated IHH expression whereas the PTH upregulated IHH expression and partly rescued the suppression of IHH expression resulting from endogenous PTH deficiency. Smo and Gli1, two downstream target genes of IHH, exhibited the same tendency as the expression of IHH. Moreover, we found that PTH1-34 elevated the expression of IHH, Smo, and Gli1 on ATDC cells, and cyclopamine, as a direct binding target for Smo, decreased the expression of Smo and Gli1, but not IHH. Interestingly, comparing the PTH and PTH&CYC groups, we observed that cyclopamine could almost completely impair the upregulated effect of PTH treatment on the expression of Smo and Gli1. Gli3, usually characterized as a negative regulator of HH signaling pathways, was a critical effector for IHH activity in the developing skeleton. Previous reports indicated that removal of Gli3 in IHH-null mouse embryos restored normal proliferation and maturation of chondrocytes, but only partially rescued the defects in osteoblast development and cartilage vascularization (Hilton et al., 2005). Gli3 acts as a repressor downstream of IHH during endochondral ossification (Koziel et al., 2005). Notably, we further detected the mRNA and protein expression of Gli3, which showed the opposite trend with Gli1. Our results demonstrated that Gli3 also plays a negative regulator in endochondral ossification during fracture healing. Thus, by activating IHH signaling pathway, exogenous PTH1-34 results in endochondral ossification acceleration and partially ameliorates delayed endochondral ossification caused by endogenous PTH deficiency during fracture healing.

How might PTH regulate the IHH signaling pathway at the fracture callus? It is well documented that PTH plays an important role in the anabolic effect on bone (Demiralp et al., 2002; Yu et al., 2009). As sharing common N-terminus and receptor (PTHR1) with PTHrp, PTH is assumed to have similar effects. Both of them can promote chondrocyte differentiation and enhance cartilage formation by elevating the expression of SRY-box nine box 9tbox9 (Huang et al., 2000; de Crombrugghe et al., 2001). PTH1–34 inhibits terminal differentiation of human articular chondrocytes in osteoarthritis rat model whereas PTH1-34 exerts earlier chondrocyte hypertrophy in callus of fracture model mice (Kakar et al., 2007; Chang et al., 2009). As a major signaling pathway, PTH binds to a membrane receptor PTHR1, increases the concentration of cAMP, activates PKA, and phosphorylates CREB, which binds to target genes through a cAMP response element (CRE) and activates their transcription. The transcriptional factor CREB, a member of a large family of basic leucine zipper (bZIP) domain DNA-binding proteins, has been proven to affect osteoblast differentiation by regulating the expression of osteoblast-specific genes and promoting chondrocyte hypertrophy and apoptosis, stimulating differentiation and maturation of osteoblasts by improving BMPR2 expression (Matsuo et al., 2006; Takai et al., 2007; Zhou et al., 2017). Additionally, the CREB family of activators could potentiate IHH signaling and regulate chondrocyte proliferation, which is required for endochondral bone development (Long et al., 2001). In our study, we found a significantly lower gene and protein expression of p-CREB in the calluses of the V-KO group compared to the V-WT group and a significantly higher gene and protein expression in the PTH-WT group compared to the V-WT group, which were consistent with IHH expression. Hence, we notably predict that CREB was one of the potential transcription factors for IHH promoters by bioinformatics analysis. ChIP and luciferase reporter assays further verified that CREB could activate IHH expression via directly binding to −1642 to −1649 bp of IHH promoter. Previous studies indicated that PTH actives the signaling transduction of the cAMP/PKA/CREB axis and promotes the phosphorylation of CREB, leading to activation of CREB and then further affecting the downstream gene expression (Tyson et al., 1999). Indeed, we observed that PTH could phosphorylate CREB, subsequently bind to the promoter of IHH, causing the activation of IHH signaling pathways.

**FIGURE 6:**
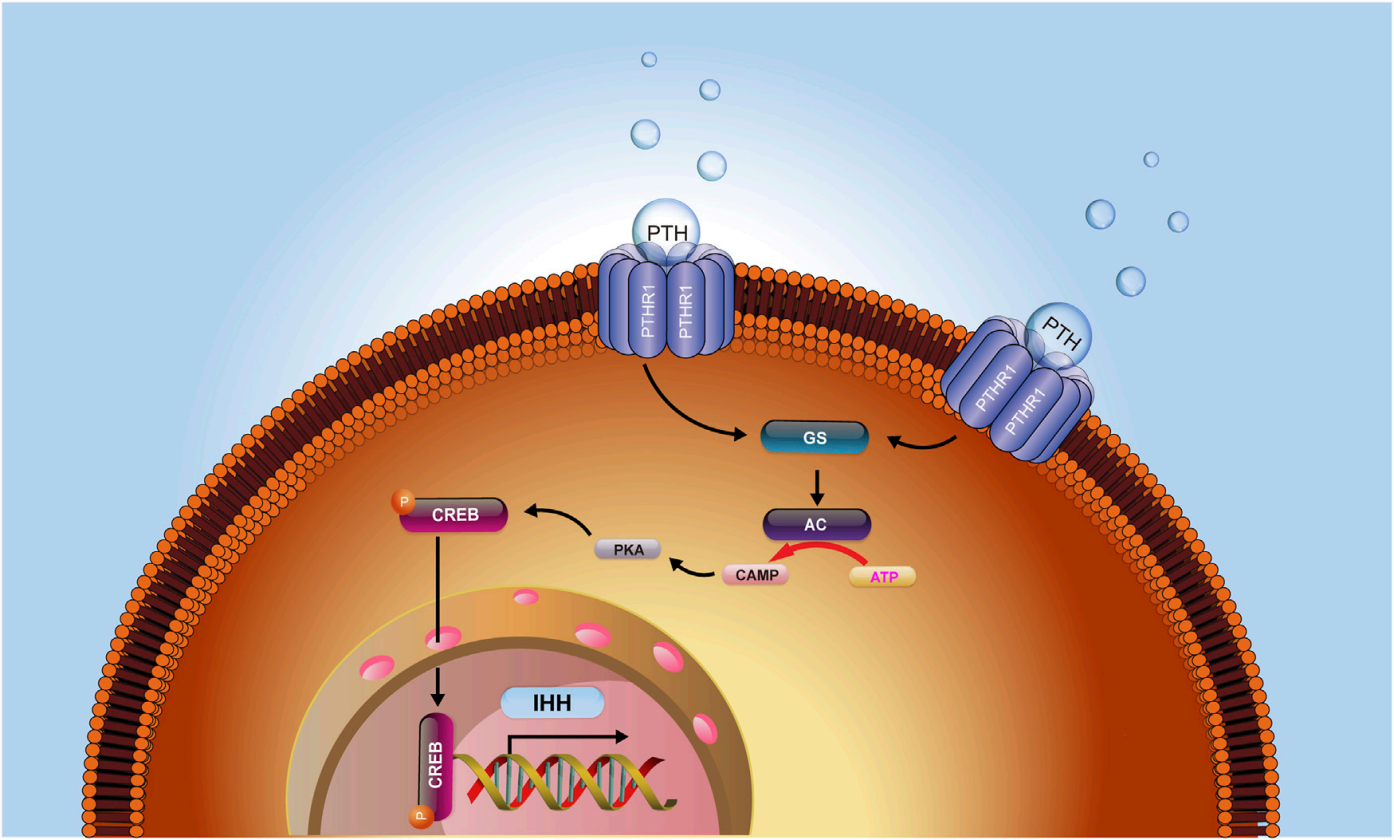
The hypothesis model depicts that PTH could increase intracellular cAMP concentrations and activate the signaling transduction of the cAMP/PKA/CREB axis, causing a subsequent increase of the level of phosphorylated CREB, which in turn activated the IHH signaling pathway. IHH further led to increased chondrogenesis in the callus and an enhanced rate of chondrocyte maturation and mineralization and ultimately accelerated the process of fracture healing.

Collectively, our current investigation identified that intermittent PTH treatment on fracture healing could increase intracellular cAMP concentrations and activate the signaling transduction of the cAMP/PKA/CREB axis, causing a subsequent increase of the level of phosphorylated CREB, which in turn activate the IHH signaling pathway. IHH further leads to increased chondrogenesis in the callus and an enhanced rate of chondrocyte maturation and mineralization and ultimately accelerated the process of fracture healing (Figure 6). Therefore, the investigation of the mechanism underlying the effects of PTH treatment on fracture repair might provide important clues to improve the understanding of fracture healing, contribute to the reveal of the influence of PTH on the biomarkers of endochondral ossification, and guide the exploration of effective therapeutic targets for fracture.

## Data Availability

The raw data supporting the conclusion of this article will be made available by the authors, without undue reservation.
