# Supplementary material for: Exogenous PTH 1-34 Attenuates Impaired Fracture Healing in Endogenous PTH Deficiency Mice via Activating Indian Hedgehog Signaling Pathway and Accelerating Endochondral Ossification
Source: Front Cell Dev Biol. 2022 Jan 5;9:750878. doi: 10.3389/fcell.2021.750878 (PMC8766796; doi:10.3389/fcell.2021.750878)
Supplement: Supplementary file 1 [file DataSheet1.docx]

**Supplementary Table S1.** Antibody used for Immunohistochemistry

| Primary Antibody | Collagen Type Ⅱ (Rabbit polyclonal) | Abcam | ab34712 | 1:200 |
| --- | --- | --- | --- | --- |
| Primary Antibody | Collagen Type Ⅹ (mouse monoclonal) | Abcam | ab49945 | 1:200 |
| Primary Antibody | PCNA (mouse monoclonal) | Abcam | ab29 | 1:10000 |
| Primary Antibody | IHH (Rabbit polyclonal) | Abcam | ab39634 | 1:200 |
| Primary Antibody | Smo (Rabbit polyclonal) | Abcam | ab72130 | 1:150 |
| Primary Antibody | Gli1(Rabbit polyclonal) | Abcam | ab151796 | 1:200 |
| Primary Antibody | Gli3(Rabbit polyclonal) | Abcam | ab6050 | 1:100 |
| Primary Antibody | PTHR1 (Rabbit polyclonal) | Abcam | ab75150 | 1:100 |
| Primary Antibody | p-CREB (Rabbit polyclonal) | Cell Signaling Technology | 9198 | 1:500 |
| Secondary Antibody | Anti-Rabbit IgG (H+L), Biotin antibody (Goat polyclonal) | Sigma-Aldrich | SAB4600006 | 1:400 |
| Secondary Antibody | Anti-Mouse IgG (H+L), Biotin antibody (Goat polyclonal) | Sigma-Aldrich | SAB4600004 | 1:400 |

**Supplementary Table S2.** Antibody used for Western blot

| Primary Antibody | Collagen Type Ⅱ (Rabbit polyclonal) | Abcam | ab34712 | 1:500 |
| --- | --- | --- | --- | --- |
| Primary Antibody | Collagen Type Ⅹ (Rabbit polyclonal) | Abcam | ab58632 | 1:300 |
| Primary Antibody | PCNA (mouse monoclonal) | Abcam | ab29 | 1:1000 |
| Primary Antibody | IHH (Rabbit polyclonal) | Abcam | ab39634 | 1:1000 |
| Primary Antibody | Smo (Rabbit polyclonal) | Abcam | ab72130 | 1:1000 |
| Primary Antibody | Gli1(Rabbit polyclonal) | Abcam | ab151796 | 1:1000 |
| Primary Antibody | Gli3(Rabbit polyclonal) | Abcam | ab6050 | 1:500 |
| Primary Antibody | PTHR1 (Rabbit polyclonal) | Sigma-Aldrich | **SAB4502493** | 1:500 |
| Primary Antibody | p-CREB (Rabbit polyclonal) | Cell Signaling Technology | 9198 | 1:1000 |
| Primary Antibody | CREB (Rabbit polyclonal) | Cell Signaling Technology | 9197 | 1:1000 |
| Primary Antibody | beta actin (mouse monoclonal) | Abcam | ab8226 | 1:1000 |
| Secondary Antibody | Anti-rabbit IgG, HRP-linked Antibody | Cell Signaling Technology | 7074 | 1:2000 |
| Secondary Antibody | Anti-mouse IgG, HRP-linked Antibody | Cell Signaling Technology | 7076 | 1:2000 |

**Supplementary Table S3.** Primers used for quantitative reverse transcription polymerase chain reaction

| Gene Name |  | 5’- Primer -3’ |
| --- | --- | --- |
| GAPDH | Forward | AGGTCGGTGTGAACGGATTTG |
|  | Reverse | TGTAGACCATGTAGTTGAGGTC |
| Col Ⅱ | Forward | A GGGAATGTCCTCTGCGATGAC |
|  | Reverse | GAAGGGGATCTCGGGGTTG |
| Col Ⅹ | Forward | TTCTGCTGCTAATGTTCTTGACC |
|  | Reverse | GGGATGAAGTATTGTGTCTTGG |
| PTHR1 | Forward | CAGGCGCAATGTGACAAGC |
|  | Reverse | TTTCCCGGTGCCTTCTCTTTC |
| PCNA | Forward | TTTGAGGCACGCCTGATCC |
|  | Reverse | GGAGACGTGAGACGAGTCCAT |
| IHH | Forward | G CTCTTGCCTACAAGCAGTTCA |
|  | Reverse | CCGTGTTCTCCTCGTCCTT |
| Smo | Forward | CCCTGCTGTGTGCTGTCTAC |
|  | Reverse | GTGTGCAACGCAGAAAGTCAG |
| Gli1 | Forward | CCAAGCCAACTTTATGTCAGGG |
|  | Reverse | AGCCCGCTTCTTTGTTAATTTGA |
| Gli3 | Forward | CACAGCTCTACGGCGACTG |
|  | Reverse | CTGCATAGTGATTGCGTTTCTTC |

Col Ⅱ indicates Collagen Type Ⅱ; Col Ⅹ, Collagen Type Ⅹ; GAPDH, glyceraldehyde-3-phosphate dehydrogenase.

**Supplementary Table S4.** Antibody used for Immunofluorescence

| Primary Antibody | Collagen Type Ⅱ (Rabbit polyclonal) | Abcam | ab34712 | 1:100 |
| --- | --- | --- | --- | --- |
| Primary Antibody | Collagen Type Ⅹ (mouse monoclonal) | Abcam | ab49945 | 1:100 |
| Primary Antibody | PCNA (mouse monoclonal) | Abcam | ab29 | 1:1000 |
| Primary Antibody | IHH (Rabbit polyclonal) | Abcam | ab39634 | 1:500 |
| Primary Antibody | Smo (Rabbit polyclonal) | Abcam | ab72130 | 1:100 |
| Primary Antibody | Gli1 (Mouse Monoclonal) | Proteintech | 66905-1-Ig | 1:400 |
| Primary Antibody | Gli3 (Rabbit polyclonal) | Abcam | ab6050 | 1:200 |
| Primary Antibody | PTHR1 (Rabbit polyclonal) | Abcam | ab75150 | 1:500 |
| Primary Antibody | p-CREB (Rabbit polyclonal) | Cell Signaling Technology | 9198 | 1:400 |
| Secondary Antibody | Goat Anti-Rabbit IgG H&L (Alexa Fluor 488)  Goat polyclonal) | Beyotime | A0423 | 1:500 |
| Secondary Antibody | Goat Anti-Mouse IgG H&L (Alexa Fluor 488)  Goat polyclonal) | Beyotime | A0428 | 1:500 |
